# Effects of 3 g of soluble fiber from oats on lipid levels of Asian Indians - a randomized controlled, parallel arm study

**DOI:** 10.1186/s12944-017-0460-3

**Published:** 2017-04-04

**Authors:** Seema Gulati, Anoop Misra, Ravindra M Pandey

**Affiliations:** 1 Diabetes Foundation (India), Safdarjung Development Area, New Delhi, India; 2 National Diabetes, Obesity, and Cholesterol Foundation (N-DOC), Safdarjung Development Area, New Delhi, India; 3 Center of Nutrition & Metabolic Research (C-NET), SDA, New Delhi, India; 4 Fortis C-DOC Center for Excellence for Diabetes, Metabolic Diseases and Endocrinology, B-16, Chirag Enclave, New Delhi, -110048 India; 5 Fortis Flt. Lt. Rajan Dhall Hospital, Vasant Kunj, New Delhi, India; 6 All India Institute of Medical Sciences (AIIMS), New Delhi, India

## Abstract

**Background:**

Cardiovascular diseases are more prevalent and severe in Asian Indians. Simple diet-based strategies are important for prevention of cardiovascular diseases.The aim of the present study was to evaluate the effects of oats consumption on lipid parameters in mildly hypercholesterolemic Asian Indians living in India.

**Methods:**

A short-term, prospective, open-labeled, randomized controlled, parallel group study was conducted. Mildly hypercholesterolemic (total cholesterol >200 mg/dL and <240 mg/dL) subjects (*n* = 80) were randomized into two groups: intervention (*n* = 40) and usual diet (*n* = 40). Sample size was calculated for a two-group parallel superiority randomized control trial. Out of 80 enrolled subjects 69 subjects completed the study; 33 in the control group and 36 in the intervention group. In the intervention group, patients were served 70 g of oats twice a day in the form of porridge and *upma* (A thick porridge from oats with seasonings and vegetables) under observation at the study site. Lipid parameters were assessed at baseline and after 4 weeks of intervention.

**Results:**

There was a reduction of 3.1% in total cholesterol levels in the control group as against 8.1% reduction in the intervention group (*p* < 0.02). Greater reductions were also seen in low-density lipoprotein cholesterol in the intervention group (11.6%) as compared to control group (4.1%, *p* < 0.04) over a period of 28 days.

**Conclusion:**

Daily consumption of 3 g of soluble fiber from 70 g of oats leads to beneficial effects on the lipid parameters, specifically total cholesterol and low-density lipoprotein cholesterol in hypercholesterolemic Asian Indians. Large scale studies over a longer period of intervention are required to further establish the cholesterol-lowering effect of oat fiber.

**Trial registration:**

The study was retrospectively registered at clinicaltrials.gov (dated: 25th Februrary.2015) with registration number NCT02376660.

## Background

Dyslipidemia is a major risk factor for cardiovascular disease (CVD). Importantly,1% increase in low-density lipoprotein cholesterol (LDL-C) levels can lead to 2% increase in CVD risk [1]. A review of ten large cohort studies reported that a decrease of 10.8 mg/dl in cholesterol concentration was associated with a decrease in risk of ischemic heart disease by 54% at age 40, 39% at age 50, 27% at age 60, 20% at age 70 and 19% at age 80 [2]. The INTERHEART study shows that, dyslipidemia appears to be the strongest contributor of acute myocardial infarction in South Asians [3].

Limited information exists regarding the changing time-trends in lipid levels and the prevalence of dyslipidemia in Asian Indians. In 1961 Padmavati et al. [4] reported mean levels of total cholesterol levels across different socio-economic groups in Delhi. Chadha et al. [5] reported 44% prevalence of hypercholesterolemia among men and 50% prevalence among women in urban Delhi during 1984–87. Indian Council of Medical Research (ICMR) study reported prevalence of hypercholesterolemia as 36.8% and 39.8% in urban Delhi and rural Haryana respectively during 1991–94 [4]. The overall prevalence of dyslipidemia in India in various studies ranges from 10% to 73% [6], depending on area of residence (rural vs. urban), socio-economic strata (high vs. middle or low), diet and physical activity patterns and age. As per Indian Council of Medical Research-India Diabetes (ICMR-INDIAB) study, highest rates of hypercholesterolemia and high LDL-C were reported from the state of Tamil Nadu, 18.3%, and 15.8% respectively [6].

The importance of nutrition in modifying the risk of CVD has been repeatedly emphasized [7–9]. Since diet forms such an integral part of an individual’s lifestyle it becomes critical to suggest changes that are easy to implement in order to ensure compliance. The need for functional foods that promote cardiovascular health, including cholesterol lowering foods, is growing. One such functional food ingredient is oat β-glucan. β -glucan is a highly viscous soluble fiber located primarily in the endosperm cell wall of oats. It is composed of glucose molecules with mixed β-(1 → 4) and β -(1 → 3) bonds. This specific chemical structure is responsible for physical properties, such as viscosity and solubility. Studies have been conducted to assess the effect of different types of fibers including fiber from oats on lipid profile of hypercholesterolemic subjects. These studies have shown that daily consumption of at least 3 g of β- glucan from oats may result in as much as a 5 to 10% reduction in total cholesterol and LDL-C concentrations [10]. A recent systematic review of 64 studies further confirms that consumption of oats is associated with reduced risk of CVD and its associated  risk factors. More than half of these studies (37/64) reported a significant reduction in total cholesterol (2–19% reduction) with about half of the studies (31/64) demonstrating significant reductions in LDL-cholesterol (4–23% reduction) over various intervention time periods [11]. In addition, Scandinavian studies have shown that adding oats to an individual’s diet may enhance overall dietary nutritional value, particularly improving vitamins, minerals and antioxidants content of the diet. Specifically, consumption of oats for six months results in measurable increase in the intake of magnesium and vitamin B1 [12].

Health claims regarding the association between cholesterol lowering and soluble fiber from oats have been approved by food standards agencies globally [United States: U.S. Food and Drug Administration [13]; Canada: Health Canada [14] and Europe: European Food Safety Authority [15].

Indian diets are high in carbohydrates, fats and sugars, which may contribute to dyslipidemia and CVD. However, there are only a few dietary intervention studies from India that focus on the prevention of dyslipidemia and other CVD risk factors [16, 17]. Since there is evidence that the prevalence of blood lipid abnormalities and other CVD risk factors varies in different ethnic groups [18] and even LDL-C-lowering effect of statins differs by ethnicity [19], hence we planned this study to understand the effects of consumption of 3 g of oat β-glucan on lipid profile of hypercholesterolemic subjects of Asian Indian ethnicity.

## Methods

### Subject population

A prospective, randomized, parallel, controlled study was approved by an independent ethics committee in accordance with the principles and requirements described in the US Code of Federal Regulations (21 CFR Part 56), Schedule Y (2005) of Indian Drugs and Cosmetic Act and Ethical Guideline for Biomedical Research on Human Participants by ICMR 2006. After obtaining informed consent eighty (80) apparently healthy, adult, male and female subjects, aged 20 to 50 years with total cholesterol values ≥200 mg/dL and <240 mg/dL were enrolled in the study. Subject factors at screening that excluded participation included: current use of lipid lowering drugs, LDL-C > 190 mg/dL or total cholesterol <200 mg/dL and >240 mg/dL or serum triglycerides >300 mg/dL, history of heavy smoking, alcoholism, pregnant or actively lactating women, and current use of weight loss diets. The primary outcome of the study was reduction in total cholesterol concentration. Sixty nine of eighty subjects (69/80) completed the study. The study was retrospectively registered at clinicaltrials.gov with registration number NCT02376660.

### Study procedure

**Fig. 1:**
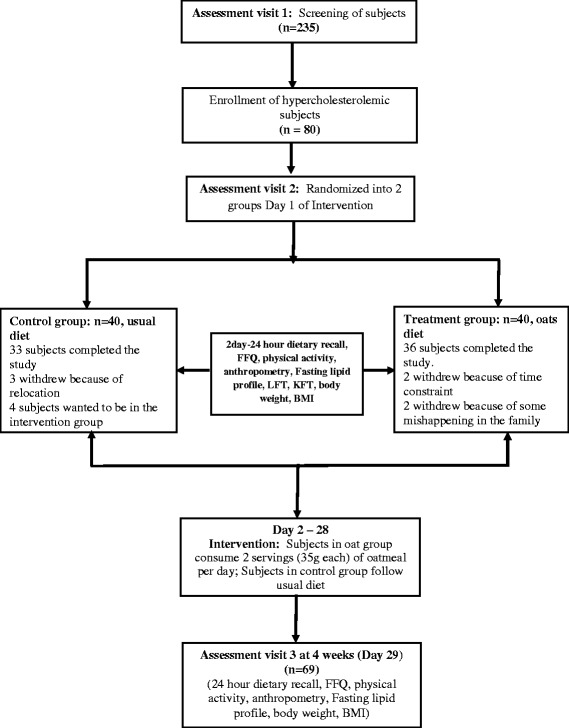
Study design

During screening pre-enrollment procedures included: obtaining informed consent, medical history review, and physical examination. Blood samples were collected for assessment of lipid profile and blood glucose levels. Urine pregnancy test, kidney and liver function tests were also completed. Subjects were enrolled in the study post-confirmation of the study inclusion and exclusion criteria. Enrolled subjects were randomized to one of the two groups; intervention or control. The intervention group received 35 g of oats twice daily (total of 70 g/day) in the form of porridge (35 g of oats) for breakfast (Quaker ® Oats, PepsiCo India) and a second serving of oats in the form of upma^1^ (35 g of oats) in the afternoon at the study site. Oats were included in the diet replacing carbohydrates from other sources as part of their ad-libitum diet. Vegetables were added in very small amount (20-30 g; two table spoons full) just to improve the palatability and acceptability of oats. The subjects in control group were already taking the vegetables. Addition of vegetables in such a small amount is not significant enough to impact the dietary composition and results. The Control group was maintained on their routine diet noted at screening. Subjects in both groups were asked to continue their usual exercise habits. After the completion of the trial, subjects wereeducated about healthy lifestyle. Subjects were provided with oat packets as their weekend breakfast and lunch/snack. Additional packets of oats (35 g each) were given for holidays and for the family (10–15 packets) to ensure better compliance. Subjects were assessed for changes in lipid parameters and anthropometry at the end of the intervention. Weekly assessments were carried out for diet, exercise, and medical history etc. along with the anthropometric evaluation as shown (Fig. 1).

**Assessment of Outcome Parameters:** Blood pressure was measured via the right arm with each participant in a sitting position according to standard protocol [20]. All assessments for anthropometry, glucose, and lipid parameters were carried out as described previously [21].

### Statistical analysis

The data were managed on an Excel 2010 spread sheet. Sample size was calculated for a two-group parallel superiority randomized control trial. A change in total cholesterol levels was taken as the primary outcome variable. Assuming mean ± SD reduction in total cholesterol in the control group as 1 ± 0.5 mmol/L and corresponding anticipated decline in the test group (oats group) as 1.4 ± 0.5 mmol/L (at least 40% more than the control group) the number of subjects required in each group to detect the above difference with 95% level of confidence and 90% power were 33 in each group. Considering a dropout rate of 20%, 40 subjects were enrolled in each arm.

Student’s *t*-test for quantitative variables was used to compare means between the two groups. Individual subject percentage change in the cholesterol concentration from baseline was computed. Mean ± SD change in cholesterol level in the two groups and their difference (effect size) was computed. Data were analyzed following the principles of intention-to-treat and per protocol analysis. For intention-to-treat analysis all the randomized subjects were included in the analysis. For missing values at 4 weeks,‘last observation carried forward’ was used to impute the values. Since baseline values were comparable for each of the lipid parameters, we used student’s *t*-test to compare the difference in the mean values at 4 weeks and reported mean difference (95% CI). For Per Protocol analysis only those subjects were considered who had completed the study. STATA v12.0 software was used for analysis. Two-sided significance was assumed at *p*-value ≤0.05.

## Results

Eighty subjects were enrolled in the study of which 11 could not complete the study due to reasons not related to the trial. The mean age of participants recruited in the control group was 31.6 ± 6.9 years and in the intervention group was 30.8 ± 6.2 years. Mean body weight of 71.2 ± 10.5 kg and 69.9 ± 9.3 kg was observed in control and intervention groups, respectively. There was no statistically significant difference between the two groups at baselines in age, gender, anthropometric, blood pressure and lipid parameters. (Tables 1 & 2) There was no significant change in body weight, body mass index (BMI), waist circumference and hip circumference, systolic and diastolic blood pressure between intrervention and control group post intervention (Table 1).

**Table 1:** Changes in anthropometric parameters and blood pressure after intervention with oats

		Per protocol				Intention-to-treat^a^			
Variable		Control (*n* = 33)	Intervention (*n* = 36)			Control (*n* = 40)	Intervention (*n* = 40)		
		Mean ± SD	Mean ± SD	Difference (95% CI)	*p* value	Mean ± SD	Mean ± SD	Difference	*p* value
ANTHROPOMETRY									
Body Weight (Kg)	Baseline	71.2 ± 10.5	69.9 ± 9.3	-	0.6	71.3 ± 11.1	70.4 ± 9.42	-	0.7
	Post intervention	71.3 ± 10.4	69.6 ± 9.5	1.6 (−3.2, 6.4)	0.5	71.3 ± 11.1	70.1 ± 9.4	1.16 (−3.4, 5.7)	0.6
Waist Circumference (cm)	Baseline	93.8 ± 7.8	92.9 ± 9.2	-	0.7	94.5 ± 8.8	93.2 ± 9.3	-	0.5
	Post intervention	93.6 ± 8.3	91.8 ± 8.3	1.8 (−2.2, 5.8)	0.4	94.3 ± 9.4	92.1 ± 8.6		0.3
Hip Circumference (cm)	Baseline	96.9 ± 6.3	96.3 ± 6.9	-	0.7	98.7 ± 9.8	96.5 ± 7.1	-	0.3
	Post intervent ion	96.9 ± 7.1	95.5 ± 7.3	1.5 (−1.9, 4.9)	0.4	98.7 ± 10.3	95.8 ± 7.3	2.9 (−0.9, 6.8)	0.2
Body Mass Index (BMI) (Kg/m2)	Baseline	25.9 ± 2.7	25.2 ± 3.5	-	0.3	26.3 ± 3.9	25.5 ± 3.6	-	0.3
	Post intervention	25.9 ± 2.7	25.1 ± 3.5	0.83 (−0.7, 2.4)	0.3	26.3 ± 3.9	25.4 ± 3.6	0.9 (−0.7, 2.6)	0.3
BLOOD PRESSURE									
Systolic Blood Pressure (mmHg)	Baseline	117.1 ± 13.7	118.2 ± 9.8	-	0.7	117.4 ± 12.9	117.8 ± 9.4	-	0.9
	Post intervention	117.4 ± 8.2	117.6 ± 8.1	−0.3 (−4.2, 3.6)	0.8	117.6 ± 8.3	117.4 ± 7.8	0.2 (−3.4, 3.8)	0.9
Diastolic Blood Pressure (mmHg)	Baseline	74.5 ± 8.7	75.8 ± 7.5	-	0.5	74.8 ± 8.4	75.6 ± 7.2	-	0.6
	Post intervention	74.7 ± 5.01	75.3 ± 6.2	−0.6 (−3.3, 2.1)	0.6	74.9 ± 5.3	75.2 ± 6.1	−0.3 (−2.8, 2.3)	0.8

^a^Subjects who were not available post intervention, pre-intervention values were carried forward as post intervention values assuming that intervention did not have an effect on them

**Table 2:** Changes in lipid parameters after intervention with oats

		Per protocol				Intention-to-treat^a^			
Variable		Control (*n* = 33)	Intervention (*n* = 36)			Control (*n* = 40)	Intervention (*n* = 40)		
		Mean ± SD	Mean ± SD	Difference (95% CI)	*p* value	Mean ± SD	Mean ± SD	Difference	*p* value
LIPID PARAMETER									
Total Cholesterol (mg/dL)	Baseline (*n* = 33)	217.2 ± 11.4	217.3 ± 10.9	-	0.8	218.1 ± 11.9	217.1 ± 10.5	-	0.7
	Post intervention	208.7 ± 24.3	197.4 ± 21.7	11.3 (0.18, 22.3)	0.04*	210.9 ± 23.3	199.2 ± 21.2	11.8 (1.8, 21.7)	0.02*
	% Reduction	4.0 ± 9.0	9.0 ± 9.7	−5.0 (−9.5, −.5)	0.03	3.3 ± 8.3	8.1 ± 9.6	−4.8 (−8.8, −.8)	0.01*
Low-Density Lipoprotein Cholesterol (mg/dL)	Baseline	145.5 ± 17.7	144.4 ± 15.7	-	0.8	144.9 ± 16.4	145.1 ± 15.4	-	0.9
	Post intervention	137.5 ± 28.4	124.9 ± 20.2	12.5 (0.8, 24.3)	0.04*	138.4 ± 25.9	127.6 ± 21.0	10.8 0.29, 22.3)	0.04*
	% Reduction	5.1 ± 17.9	12.9 ± 14.0	−7.8 (−15.5, −.11)	0.04*	4.1 ± 16.3	11.6 ± 13.8	−7.4(−14.1, −0.7)	0.03*
Very Low-Density Lipoprotein Cholesterol (mg/dL)	Baseline	31.5 ± 11.3	32.4 ± 11.9	-	0.8	32.4 ± 10.9	32.7 ± 12.5		0.9
	Post intervention	34.1 ± 16.1	32.9 ± 13.4	1.2 (−5.9, 8.3)	0.7	43.5 ± 15.1	33.2 ± 13.8	1.4 (−5.1, 7.8)	0.7
	% Reduction	−21.8 ± 73.0	−7.1 ± 39.9	−14.7 (−42.7, 13.2)	0.3	−18.0 ± 66.7	−6.3 ± 37.9	−11.6 (−35.7, 12.5)	0.3
Serum Triglycerides (mg/dL)	Baseline	157.6 ± 56.2	161.9 ± 59.4	-	0.8	162.2 ± 54.7	163.6 ± 62.5	-	0.9
	Post intervention	170.3 ± 80.6	164.4 ± 67.2	5.9 (−29.6, 41.5)	0.7	172.7 ± 75.2	165.8 ± 69.2	6.8 (−25.3, 38.9)	0.7
	% Reduction	−21.8 ± 73.0	−7.1 ± 39.9	−14.7 (−42.7, 13.2)	0.3	−18.0 ± 66.7	−6.4 ± 37.9	−11.6(−35.8, 12.5)	0.34
High-Density Lipoprotein Cholesterol (mg/dL)	Baseline	39.1 ± 8.3	41.5 ± 12.5	-	0.4	39.6 ± 8.1	41.2 ± 12.1		0.5
	Post intervention	37.1 ± 7.2	39.4 ± 9.5	−2.4 (−6.5, 1.7)	0.2	37.9 ± 7.3	39.3 ± 9.3	−1.5 (−5.2, 2.3)	0.4
	% Reduction	3.8 ± 15.1	3.3 ± 14.1	0.5(−6.5, 7.5)	0.9	3.1 ± 13.7	2.9 ± 13.3	0.2 (−5.8, 6.2)	0.9

^a^Subjects who were not available post intervention, pre-intervention values were carried forward as post intervention values assuming that intervention did not have an effect on them

*p < 0.05: statistically significant

As per intention-to-treat analysis, there was a reduction in the mean total cholesterol levels from 218.1 ± 11.9 mg/dL to 210.1 ± 11.9 mg/dL (3.3%) and 217.1 ± 10.5 mg/dL to 199.2 ± 21.2 mg/dL (8.1%) after intervention in the control and intervention groups, respectively. The difference between the groups was statistically significant post intervention (*p* < 0.01).

Reductions in LDL-C levels exhibited a similar trend. A reduction in mean LDL-c from 144.9 ± 16.4 mg/dL to 138.4 ± 25.9 mg/dL (4.1%) and from 145.1 ± 15.4 mg/dL to 127.6 ± 21.0 mg/dL (11.6%) was observed in the control and the intervention groups post intervention respectively. The reduction in LDL-C was significantly higher in the intervention group than the control group. (Table 2) There was no significant change in very low-density lipoprotein cholesterol (VLDL-C), serum triglycerides and high- density lipoprotein cholesterol (HDL-C) post intervention. However, the trends for serum triglycerides and VLDL-C reduction were better in the intervention group than in the control group (Table 2). As per Per Protocol analysis the data of 69 subjects showed a significant reduction in mean total cholesterol levels (*p* < 0.04) and LDL-C levels (*p* < 0.04) post intervention as well. There was reduction of 4.0% in total cholesterol levels in the control group as against 9% reduction in total cholesterol in the intervention group. The LDL-C reduction was also higher in intervention group; 12.9% as compared to control group; 5.1% (Table 2). There was no significant change in VLDL-C, serum triglycerides and HDL-C post intervention.

## Discussion

The present study shows the cholesterol lowering property of dietary oats in the Asian Indians. To our knowledge this is the first study on oats conducted in Indian population.

A meta-analysis of 25 studies on oats shows that 1 g of soluble fibre from oats leads to 1.42 mg/dL and 1.23 mg/dL reductions in total cholesterol and LDL-C levels respectively [22]. Two possible mechanisms have been proposed to explain the decrease observed in total cholesterol. One hypothesis is that soluble fibre decreases absorption of cholesterol and fatty acids and increases bile acid excretion. It has also been proposed that soluble fibres reduce cholesterol synthesis by production of short chain fatty acids that affect lipid metabolism [23]. In a published animal study an increased amount of bile acids was observed in the intestinal, caecal and colonic contents of the test groups [24]. Another recently conducted animal study hypothesized the possible up-regulation of CYP7A1 and CYP8B1 (bile acid producing hepatic enzymes) thus further supporting the bile acid related cholesterol reducing mechanism of oats [25].

Another recent area of research is the factors that influence the bioactivity of oats. One of the major factors is the molecular weight of β- glucan. Primitive findings of a study showed that consumption of 3 g high molecular weight (2,250,000 g/mol), 4 g medium molecular weight (850,000 g/mol) and 3 g medium molecular weight (530,000 g/mol) oat β- glucan daily for 4 weeks showed significant reductions in LDL-C (4.8 to 6.5% reductions) [26]. Thus, it is important to note that all oats may not have the same cholesterol lowering property as it has been proposed but not proven that high molecular weight of β-glucan increases viscosity of intestinal content. However, the reductions seen in this study, i.e. 8.1% reduction in total cholesterol and 11.6% in LDL-C are comparable with the reductions observed in the published studies [11, 26]. It was also observed that after intervention with oats, not only did the total cholesterol levels dropped significantly in the intervention arm but also the number of subjects having total cholesterol within the normal range i.e. < 200 mg/dl was higher in intervention arm, 19 out of 36 (52.8%) subjects as against control arm, where 12 out of 33 subjects (36.4%) had total cholesterol levels within the normal range. It has been observed that oats may be beneficial for insulin sensitivity and glycemic parameters, thus controlling serum triglycerides [27]. These actions may have resulted in stable serum triglyceride levels in the oats group vs. non-significant slight increase in the control group. A longer duration study is required to understand the effect of oats on triglycerides and VLDL-cholesterol.

## Conclusion

The study findings show that a small lifestyle modification such as introduction of soluble fibre from oats can have a significant lowering effect on total cholesterol levels in hypercholesterolemic Asian Indian subjects. However, large scale studies over a longer period of intervention are required to further establish the cholesterol lowering effect of oat fiber.

## Abbreviations

CFR: Code of Federal Regulations
CVD: cardiovascular disease
CYP7A1: Cholesterol 7-alpha-monooxygenase
CYP8B1: Cytochrome P450
HDL-C: High- density lipoprotein cholesterol
ICMR: Indian Council of Medical Research
ICMR-INDIAB: Indian Council of Medical Research-India Diabetes
LDL-C: low-density lipoprotein cholesterol
SD: Standard deviation
VLDL-C: Very low-density lipoprotein cholesterol

## Footnotes

1 A thick porridge from oats with seasonings and vegetables added to it during the cooking.
